# Susceptibility of *Exopalaemon carinicauda* to the Infection with Shrimp Hemocyte Iridescent Virus (SHIV 20141215), a Strain of Decapod Iridescent Virus 1 (DIV1)

**DOI:** 10.3390/v11040387

**Published:** 2019-04-25

**Authors:** Xing Chen, Liang Qiu, Hailiang Wang, Peizhuo Zou, Xuan Dong, Fuhua Li, Jie Huang

**Affiliations:** 1 Laboratory for Marine Fisheries Science and Food Production Processes, National Laboratory for Marine Science and Technology (Qingdao); Key Laboratory of Maricultural Organism Disease Control, Ministry of Agriculture and Rural Affairs; Qingdao Key Laboratory of Mariculture Epidemiology and Biosecurity; Yellow Sea Fisheries Research Institute, Chinese Academy of Fishery Sciences, Qingdao 266071, China; chenxing910520@163.com (X.C.); qiuliang@ysfri.ac.cn (L.Q.); whl846130@126.com (H.W.); zoupz1992@163.com (P.Z.); dongxuan@ysfri.ac.cn (X.D.); 2 College of Fisheries and Life Science, Shanghai Ocean University, Shanghai 201306, China; 3 Key Laboratory of Experimental Marine Biology, Institute of Oceanology, Chinese Academy of Sciences, Qingdao 266071, China; fhli@ms.qdio.ac.cn

**Keywords:** SHIV, DIV1, *Decapodiridovirus*, *Exopalaemon carinicauda*, susceptibility, host, ISDL

## Abstract

In this study, ridgetail white prawns—*Exopalaemon carinicauda*—were infected per os (PO) with debris of *Penaeus vannamei* infected with shrimp hemocyte iridescent virus (SHIV 20141215), a strain of decapod iridescent virus 1 (DIV1), and via intramuscular injection (IM with raw extracts of SHIV 20141215. The infected *E. carinicauda* showed obvious clinical symptoms, including weakness, empty gut and stomach, pale hepatopancreas, and partial death with mean cumulative mortalities of 42.5% and 70.8% by nonlinear regression, respectively. Results of TaqMan probe-based real-time quantitative PCR showed that the moribund and surviving individuals with clinical signs of infected *E. carinicauda* were DIV1-positive. Histological examination showed that there were darkly eosinophilic and cytoplasmic inclusions, of which some were surrounded with or contained tiny basophilic staining, and pyknosis in hemocytes in hepatopancreatic sinus, hematopoietic cells, cuticular epithelium, etc. On the slides of in situ DIG-labeling-loop-mediated DNA amplification (ISDL), positive signals were observed in hematopoietic tissue, stomach, cuticular epithelium, and hepatopancreatic sinus of infected prawns from both PO and IM groups. Transmission electron microscopy (TEM) of ultrathin sections showed that icosahedral DIV1 particles existed in hepatopancreatic sinus and gills of the infected *E. carinicauda* from the PO group. The viral particles were also observed in hepatopancreatic sinus, gills, pereiopods, muscles, and uropods of the infected *E. carinicauda* from the IM group. The assembled virions, which mostly distributed along the edge of the cytoplasmic virogenic stromata near cellular membrane of infected cells, were enveloped and approximately 150 nm in diameter. The results of molecular tests, histopathological examination, ISDL, and TEM confirmed that *E. carinicauda* is a susceptible host of DIV1. This study also indicated that *E. carinicauda* showed some degree of tolerance to the infection with DIV1 per os challenge mimicking natural pathway.

## 1. Introduction

The iridescent virus family *Iridoviridae* contains large icosahedral double-stranded DNA viruses, which is divided into two subfamilies (i.e., *Alphairidovirinae* and *Betairidovirinae*) and composed of five known genera: *Lymphocystivirus*, *Megalocytivirus*, *Ranavirus*, *Chloriridovirus*, and *Iridovirus*. Of them, the genera *Lymphocystivirus*, *Megalocytivirus*, and *Ranavirus* belong to subfamily *Alphairidovirinae*, and *Chloriridovirus* and *Iridovirus* belong to subfamily *Betairidovirinae* [1,2,3]. Iridescent viruses of *Alphairidovirinae* lead to a high mortality rate in significant fish and amphibians [3], while iridescent viruses of *Betairidovirinae* infect insects and crustaceans. The first discovery of a possible iridescent virus in crustaceans was published in 1993 [4]. In the same year, Lightner and Redman reported another suspected iridescent virus in penaeid shrimp *Protrachypene precipua* [5]. Subsequently, Miao et al. [6] reported that a suspected iridescent virus was observed in lymphoid cell cultures from the Chinese white shrimp *Penaeus chinensis* in 1999. Several years later, Tang et al. [7] identified an iridoviru—i.e., Sergestid iridovirus (SIV)—diseased sergestid shrimp *Acetes erythraeus*, which is a likely causative agent evidenced through in situ hybridization and PCR test. Xu et al. [8] reported *Cherax quadricarinatus* iridovirus (CQIV) isolated from diseased red claw crayfish *Cherax quadricarinatus*. Qiu et al. [9] identified an iridescent virus named shrimp hemocyte iridescent virus (SHIV), which was isolated from farmed *Penaeus vannamei* in 2014 and also detected in *P. chinensis* and *Macrobrachium rosenbergii*. In March 2019, the Executive Committee of the International Committee on Taxonomy of Viruses (ICTV) approved the proposal made by Chinchar et al. [10] that proposed a new species of decapod iridescent virus 1 in a new genus *Decapodiridovirus* to include SHIV 20141215 and CQIV CN01 as two isolates.

The range of susceptible hosts is an important part of epidemiology and pathogen ecology. It is also of significant concerned for international trade and control of the disease. In 2014, the World Organization for Animal Health (OIE) has adopted a chapter in *The Aquatic Animal Health Code* to provide the criteria for determining susceptibility of aquatic animal species to infection with a specific pathogen with emphasis on that the route of transmission is consistent with natural pathways for the infection [11]. Ridgetail white prawn—*Exopalaemon carinicauda*—is one of the major economic crustaceans in China, which can propagate all year round, and is naturally distributes around the coast of Yellow Sea and Bohai Sea of China [12,13]. *E. carinicauda* accounts for one-third of the total yields of polyculture ponds in the eastern China [12]. It is also a species of wild prawns and commonly exists in the ponds farming *M. rosenbergii* or penaeid shrimp. To date, there is no report pointing out if *E. carinicauda* can be infected with iridescent viruses. Owing to the common existence of *E. carinicauda* in intensive shrimp farming ponds, verifying its susceptibility to DIV1 is a prerequisite for evaluation of the potential transmission routes and possible reservoirs of DIV1 in the nature. As there is no crustacean cell line available, bioassays with susceptible species are the only way to study the infectivity of crustacean viruses. Establishment of infection model with susceptible animals will provide approaches for crustacean virological studies. In this study, *E. carinicauda* were challenged per os to mimic natural infection according to the OIE standards, while intramuscular injection was used as a positive control. A combination of TaqMan real-time PCR, histopathological observation, in situ DIG-labeling-loop-mediated DNA amplification (ISDL), and ultra-thin transmission electron microscopy were used to confirm the infection with DIV1 in *E. carinicauda*.

## 2. Materials and Methods

All protocols for animal breeding, handling and sampling were approved by the Animal Care and Ethics Committee, Yellow Sea Fisheries Research Institute, Chinese Academy of Fishery Sciences. Efforts were made to provide a comfortable living environment for the animals and to minimize the suffering of animals during the sampling process, according to recommendations proposed by the European Commission (1997). The study was carried out in accordance with the approved protocol. All the methods were applied in accordance with relevant guidelines.

### 2.1. Animals

Specific-pathogen-free (SPF) ridgetail white prawns *E. carinicauda*, (3.6 ± 0.4) cm in body length, were reared and bred in the Key Laboratory of Experimental Marine Biology, Institute of Oceanology, Chinese Academy of Sciences. Prawns *E. carinicauda* were cultivated in a 40 L plastic tanks containing 20 L seawater of 30 ppt in salinity with 90% daily exchange rate, at a water temperature of 27 °C, with continuous aeration, and fed three times per day with the formula feed for a week in our wet lab. For multiplication of DIV1, 30 healthy white leg shrimp *P. vannamei* of (10.2 ± 0.8) cm in body length were purchased from a shrimp farm in Weifang of Shandong Province and then held in 40 L seawater of 30 ppt salinity with same management above mentioned.

Before and after the challenge testing, the prawns and the shrimp were sampled and examined for potential pathogens, including white spot syndrome virus (WSSV), taura syndrome virus (TSV), yellow head virus (YHV), infectious hypodermal and hematopoietic necrosis virus (IHHNV), and acute hepatopancreatic necrosis disease-causing *Vibrio parahaemolyticus* (*Vp*_AHPND_), using the nested PCR or TaqMan probe based quantitative real-time PCR (TaqMan qPCR) methods [14], covert mortality nodavirus (CMNV) using TaqMan qPCR methods [15], and DIV1 using nested PCR [16].

### 2.2. Preparation of Viral Inoculum

To prepare material for the challenge test, 30 healthy shrimp *P. vannamei* were infected by feeding with DIV1 (strain SHIV 20141215) infected tissue, which was derived from diseased *P. vannamei* collected at a farm in Zhejiang Province in December 2014 [9]. After 5 days post-infection (dpi), 2.5 g cephalothoraxes (removing tergites) were taken from DIV1-infected shrimp and subsequently homogenized in 40 mL pre-cooled PPB-Tris (376.07 mM NaCl, 6.32 mM K_2_SO4, 6.4 mM MgSO_4_, 14.41 mM CaCl_2_, and 50 mM Tris-HCl, pH 6.5–8.0) [17]. The suspension was centrifuged at 10,000 rpm for 10 min at 4 °C. The pellet was resuspended in 25 mL PPB-Tris by homogenization and re-centrifuged. The supernatants from two steps were merged and filtered through a 500-mesh sieve and a 0.45 µm filter. The suspension was sampled to detect various pathogens for exclusion of contamination by other viruses and bacteria. DIV1 dose in the suspension was quantified using TaqMan qPCR method [16]. For the challenge test via intramuscular injection, an inoculum containing 10^4^ copies/µL DIV1 was prepared by diluting the suspension with PPB-His (replace 50 mM Tris-HCl with 50 mM histidine in PPB-Tris) [17], and then the inoculum was dispensed in 100 µL aliquots before stored at −80 °C.

### 2.3. Challenge Tests

Challenge tests were performed with healthy *E. carinicauda* in four groups, including intramuscular injection (IM), per os (PO), control of intramuscular infection (CIM), and control of per os (CPO). Each group had three replicates with 10 individual prawns per replicate. Each prawn in the IM group was injected with 10 µL of viral inoculum (~10^5^ copies). Prawns in the PO group were fed with total 3 g debris of DIV1-infected *P. vannamei* tissue (with a viral dose of about 10^10^ copies/g). Prawns in the CIM group and the CPO group were injected with 10 µL sterile PPB-His and commercial formula feed, respectively. After the challenge operation, the rearing conditions were kept as same as those prior to the challenge.

Significance analysis of cumulative mortalities between any two groups was carried out using the *t*-test for homoscedasticity hypothesis of two group samples with the add-in tool of data analysis in Microsoft^®^ Excel^®^ 2016 MSO 64-bit.

For evaluation of the pathogenic model in the infection groups, mortality data were nonlinearly regressed following the three-parameter sigmoid equation

(1) $$M_{\left(t\right)}=\frac{a}{1+e^{-k\left(t-t_{0}\right)}},$$

which formulates the change of *M*_(*t*)_ (cumulative mortality) at *t* (time, dpi) determined by parameters of *a* (the maximum mortality), *t*_0_ (the time to half of the maximum mortality), and *k* (the instant incidence rate). The real pathogenic model shall deduct the average mortality of control groups.

### 2.4. Detection of DIV1 Using TaqMan-Probe-Based Real-Time Quantitative PCR (TaqMan qPCR)

Total DNA were extracted from the hepatopancreas of samples stored at −20 °C using a TIANamp Marine Animals DNA Kit (Tiangen, Beijing, China). TaqMan qPCR, using the total DNA as template, was conducted as described previously [16] to detect and quantify DIV1. The forward and reverse primers were SHIV-F 5’-AGG AGA GGG AAA TAA CGG GAA AAC-3’, and SHIV-R 5’-CGT CAG CAT TTGGTT CAT CCA TG-3’, respectively. The TaqMan probe (5’-CTG CCC ATC TAA CAC CAT CTC CCG CCC-3’) was labeled with 5’-6-carboxyfluorescein (FAM) and 3’-carboxytetramethylrhodamine (TAMRA). Each PCR mixture in 20 µL contained 10 µL 2× FastStart Essential DNA Probes Master (Roche, Indianapolis, IN, USA), 500 nM primer each, 200 nM probe and 100 ng total DNA template. The program started by an initial denaturation at 95 °C for 10 min, followed by 40 cycles at 95 °C for 10 s and 60 °C for 30 s. The amplification and date analysis were carried out in a CFX-96 Quantitative Fluorescence Instrument (BioRad, Hercules, California, USA).

### 2.5. Histopathological Examination

The cephalothoraxes of *E. carinicauda*, sampled during the challenge tests, were fixed with Davison’s AFA fixative (DAFA) for 24 h and processed for sectioning and hematoxylin and eosin (H&E) staining as described by Bell & Lightner [18]. The histological sections were analyzed and photographed under a light microscopy system (Eclipse 80i, Nikon, Tokyo, Japan).

### 2.6. Loop-Mediated Isothermal Amplification (LAMP)

A set of specific primers, composed of FIP, BIP, LF, LB, F3, and B3, for LAMP detection of DIV1 was designed to target the gene of the second largest subunit of DNA-directed RNA polymerase II in the DIV1 genomic sequence (GenBank accession No. MF599468), using PrimerExplorerV4 (http://primerexplorer.jp/elamp4.0.0/index.html). These primers compared with the database in NCBI (http://blast.ncbi.nlm.nih.gov/Blast.cgi) to analyze sequence similarities. The primers were synthesized by Sangon Biotech (Shanghai, China). Each LAMP mixture contained 1.6 µM each of inner primers FIP and BIP, 0.8 µM each of loop primers LF and LB, 0.2 µM each of outer primers F3 and B3, 1.4 mM of dNTP mix (TaKaRa, Dalian, China), 1.2 M betaine (Solarbio, Beijing, China), 25 µM calcein (Sigma, St. Louis, MO, USA), 500 µM MnCl_2_, 6 mM MgCl_2_, 8 U Bst 2.0 DNA polymerase (New England Biolabs Inc., Beverly, USA), 1× supplied buffer and the specified amount of template DNA in a final volume of 25 µL. The procedure was 60 cycles for 60 °C, following 5 min at 85 °C on the CFX-96 Quantitative Fluorescence Instrument (BioRad, Hercules, CA, USA) using calcein fluorescent channel. Detection specificity of the LAMP primers were examined using 100 ng of the total DNA extracted from uninfected prawns and shrimp infected with other pathogens, including WSSV, *Vp*_AHPND_, IHHNV, and EHP.

### 2.7. In Situ DIG-Labeling-Loop-Mediated DNA Amplification (ISDL)

ISDL followed the method published by Jitrakorn et al. [19] with some modification to target DIV1. Paraffin sections were dewaxed and rehydrated according to the normal DIG-labeled in situ hybridization method [18,19,20]. Rehydrated slides were added with ddH_2_O and denatured on a 100 °C heating block for 2 min, then subsequently placed in a wet box. Total of 150 µL LAMP mixture, which has the same constituents as described in paragraph 2.6, except that the dNTP mix was supplemented with 0.1 mM digoxigenin-11-dUTP (DIG-labeled dUTP) and—without template, calcein, and MnCl_2_—were added dropwise to each slide. The slides were horizontally incubated at 65 °C for 60 min, followed by 85 °C for 5 min. Subsequent steps were performed in accordance with the post-hybridization steps of a normal in situ hybridization [20]. Tissue sections of healthy prawns were used as the control.

### 2.8. Transmission Electron Microscopy with Ultrathin Sections

For transmission electron microscopy (TEM) with ultrathin sections, samples were prepared as previous described [21,22]. Briefly, tissues of hepatopancreas, muscle, pereiopods, uropods, and gills of infected *E. carinicauda* were placed in TEM fixative (2% paraformaldehyde, 2.5% glutaraldehyde, 160 mM NaCl and 4 mM CaCl_2_ in 200 mM PBS, pH 7.2) and cut rapidly into ~1 mm^3^ pieces with scalpels, fixed for 1 h at room temperature, and then post-fixed with OsO_4_. Specimens were embedded in Spurr’s plastic and dyed with uranyl acetate and lead citrate. Ultrathin sections were prepared on collodion-coated grids by the Equipment Center of the Medical College, Qingdao University (Qingdao, China). All grids were examined under a JEM-1200 electron microscope (JEOL, Japan) operating at 80–100 kV.

## 3. Results

### 3.1. Clinical Signs and Cumulative Mortality

**Figure 1:**
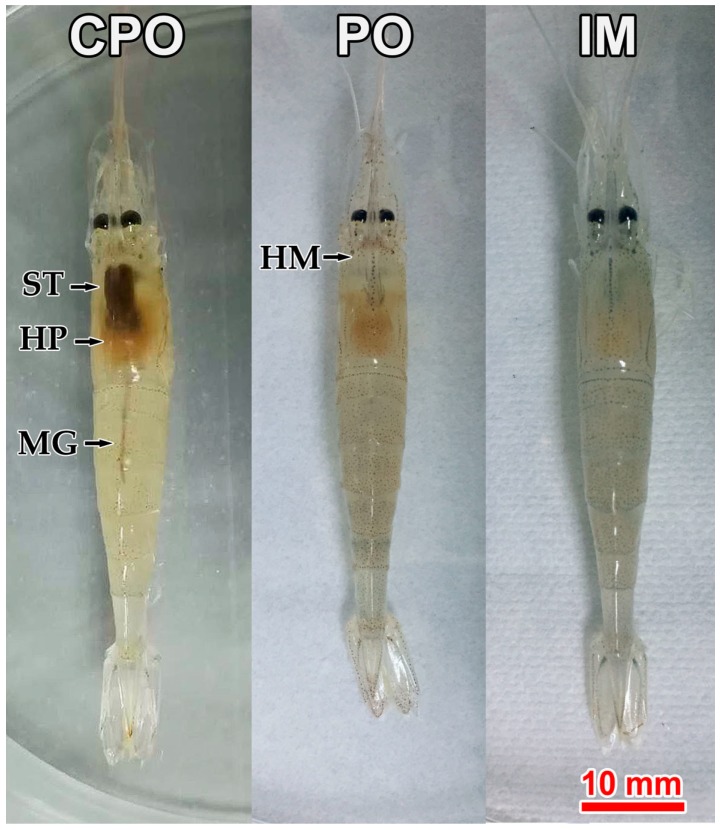
Gross signs of prawns *Exopalaemon carinicauda* in different groups of the challenge test. CPO: Prawn in per os control group; PO: Prawn in per os group; IM: Prawn in intermuscular injection group. Solid arrows indicate stomach (ST), hepatopancreas (HP), midgut (MG), and hematopoietic tissue (HM). Red bar = 10 mm.

The challenge test lasted for 15 days. After 3 dpi, prawns in both intramuscular (IM) and per os (PO) groups showed clinically symptoms, including empty stomach and gut and pale hepatopancreas. Additionally, the hematopoietic tissue at the base of rostrum showed slight cloudy white (Figure 1, IM and PO); while prawns in control groups (i.e., CPO and CIM) displayed normal gross signs, including feed-filled stomach and gut and light brown hepatopancreas. However, the hematopoietic tissue was not visible (Figure 1, CPO).

**Figure 2:**
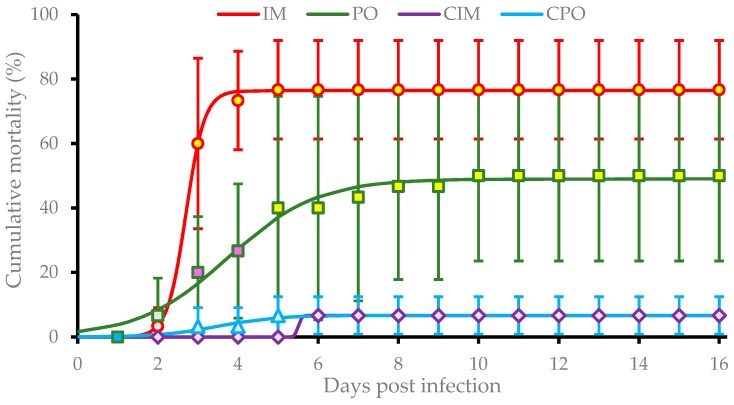
Cumulative mortalities of *Exopalaemon carinicauda* in the challenge test. IM group, prawns were challenged with filtrated viral suspension via intermuscular injection; PO group, prawns were fed with tissues of DIV1-infected *P. vannamei*; CIM group, prawns were injected with sterile PPB-His buffer; CPO group, prawns were fed with commercial feed. Cumulative mortalities are shown as means of data from three replicates for each experimental group (each replicate contained 10 individuals). The mean points with same color indicate no significant difference (*P* > 0.05), and the mean points with different colors indicate a significant difference (*P* < 0.05). Overall analysis indicated there are very significant differences among IM, PO, and the two controls (*P* < 0.01). The curves were drawn based on the nonlinear regression following the three-parameter sigmoid equation.

Prawns in IM and PO groups suffered from a rapid increase in mortality during the period between 2 dpi and 5 dpi. The average cumulative mortality stabilized at (76.7 ± 18.3)% and (50.0 ± 26.5)% in the IM group after 5 dpi and PO group after 10 dpi, respectively. These rates were both significantly higher (*P* < 0.01) than that of CIM and CPO groups which both had an average mortality of (6.7 ± 5.8)%. Additionally, the cumulative mortality of the IM group was significantly higher (*P* < 0.05) than that of the PO group at 3 dpi and 4 dpi (Figure 2). Significance analysis of the overall data of four groups showed significant differences among IM, PO, and control groups (*P* < 0.01).

Based on the nonlinear regression following the three-parameter sigmoid equation, the pathogenic models of infection with DIV1 on *E. carinicauda* via IM and PO were

(2) $$M_{IM\left(t\right)}=\frac{70.8\pm 0.2}{1+e^{-\left(4.76\pm 2.49\right)\left[t-\left(2.64\pm 0.21\right)\right]}}$$

(3) $$\mathrm{and}\;M_{PO\left(t\right)}=\frac{42.5\pm 1.2}{1+e^{-\left(0.82\pm 0.51\right)\left[t-\left(3.83\pm 0.87\right)\right]}},$$

respectively. The functions (2) and (3) indicated the infection of *E. carinicauda* with DIV1 via IM caused (70.8 ± 0.2)% maximum cumulative mortality, while the infection PO caused (42.5 ± 1.2)% maximum cumulative mortality. The former reached the half level of the maximum mortality at (2.64 ± 0.21) dpi with an instant incidence rate at (4.76 ± 2.49)/day; and the latter did at (3.83 ± 0.87) dpi with an instant rate at (0.82 ± 0.51)/day.

### 3.2. TaqMan qPCR Detection

**Figure 3:**
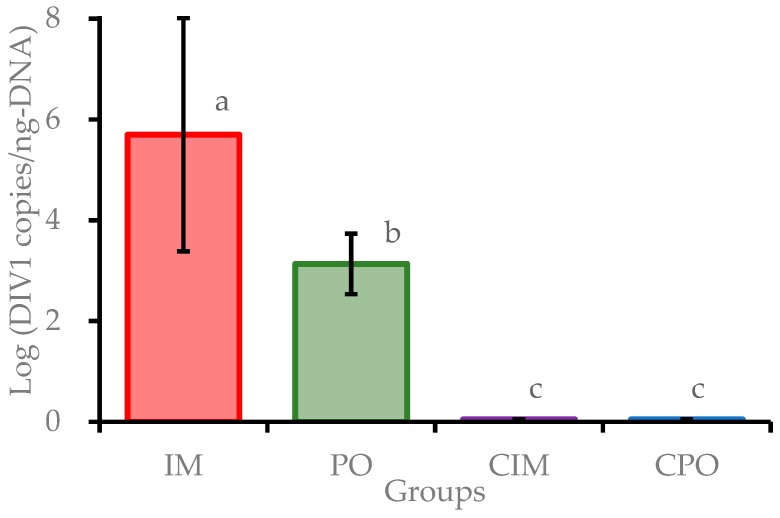
Decapod iridescent virus 1 (DIV1) loads in *Exopalaemon carinicauda* from the challenge test. Different letters above the bars indicate significant difference (*P* < 0.01). IM: prawn group challenged var intramuscular injection, PO: prawn group challenged per os; CIM: control group of injected with sterile buffer; CPO: control group fed with commercial feed. CIM and CPO were negative.

Moribund prawns in IM and PO groups were sampled throughout the experimental duration, and all surviving prawns in each group were collected at the end of the challenge experiment. All samples were examined for potential pathogens, using the TaqMan qPCR method. The DIV1-positive rates were 80.0% (24/30) and 46.7% (14/30) in the IM group and PO group, respectively. The results showed that all DIV1 negative prawns of the IM and PO groups survived and all prawns of CIM and CPO groups were DIV1 negative. The tests of *Vp*_AHPND_, IHHNV and WSSV were negative for all samples. The logarithmic DIV1 loads (in copies/ng-DNA) in positive prawns of the IM and PO groups were 5.65 ± 2.31 and 3.08 ± 0.60, respectively. The viral loads in positive prawns of the IM group were significantly higher (*P* < 0.01) than those of the PO group (Figure 3). The loads of DIV1 in 21 moribund and dead prawns of the IM group were detected to reach (4.20 ± 3.88) × 10^6^ copies/ng-DNA, while that in 3 non-clinical prawns were detected at only (5.47 ± 4.16) × 10^−1^ copies/ng-DNA. The loads of DIV1 in 7 dead prawns in the PO group during 5–7 dpi averaged at (3.63 ± 1.44) × 10^4^ copies/ng-DNA, which were much higher than that in 7 surviving prawns in the same group at (3.57 ± 3.42) × 10^2^ copies/ng-DNA (Figure S1). These results showed that prawns in the IM and PO groups were successfully infected by DIV1. In addition, the results suggested that *E. carinicauda* may have some resistance to infection with DIV1 per os mimicking a natural exposure pathway.

### 3.3. Histopathology

**Figure 4:**
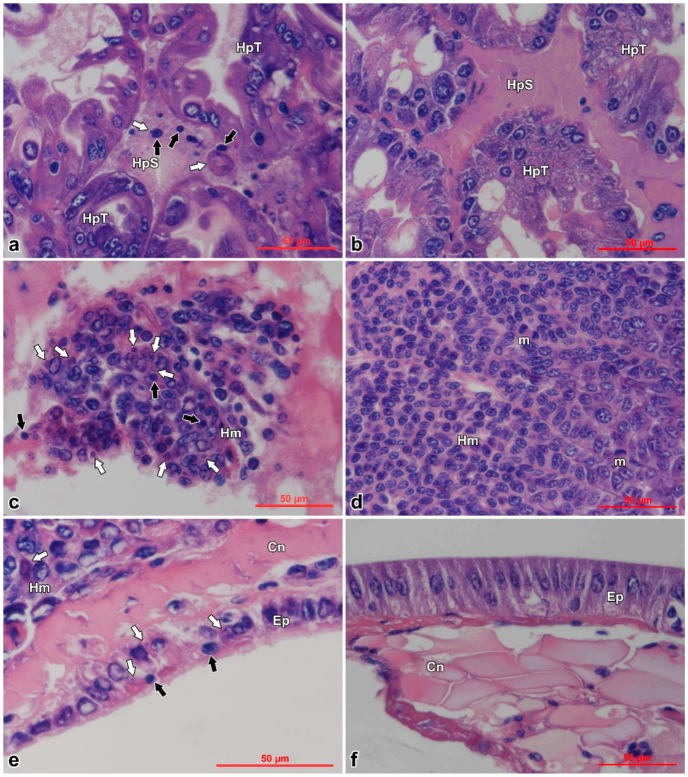
Histopathological examination of *Exopalaemon carinicauda* tissues infected with DIV1 and controls. Black arrows show karyopyknosis and white arrows show eosinophilic inclusions. Pictures (**a**,**b**) are hepatopancreas; (**c**,**d**) are hematopoietic tissues; and (**e**,**f**) are cuticular epithelium on which the cuticles were removed before dehydration. The pictures in the left column (a, c, and e) are the tissues of the infected prawn; the pictures in the right column (b, d, and f) are the tissues of the control prawn. HpT: hepatopancreatic tubule; HpS: hepatopancreatic sinus; Hm: hematopoietic tissue; Ep: epithelium; Cn: connective tissue; m: mitotic phase. Bar = 50 µm.

Histopathological examination of moribund prawns showed nuclear pyknosis and acidophilic inclusions in hemocytes of hepatopancreatic sinus (Figure 4a), hemocytoblasts of hematopoietic tissues (Figure 4c), and cuticular epithelium (Figure 4e); while the control prawns appeared normal in these tissues (Figure 4b,d,f). Typical cytoplasmic and dark eosinophilic inclusions, of which some were surrounded with or contained tiny basophilic staining, appeared beside the nuclei in hematopoietic tissues, hemocytes, and epithelium. The inclusions were more easily found in the infected hematopoietic tissues (Figure 4b).

### 3.4. Primer Design and LAMP Reaction

**Figure 5:**
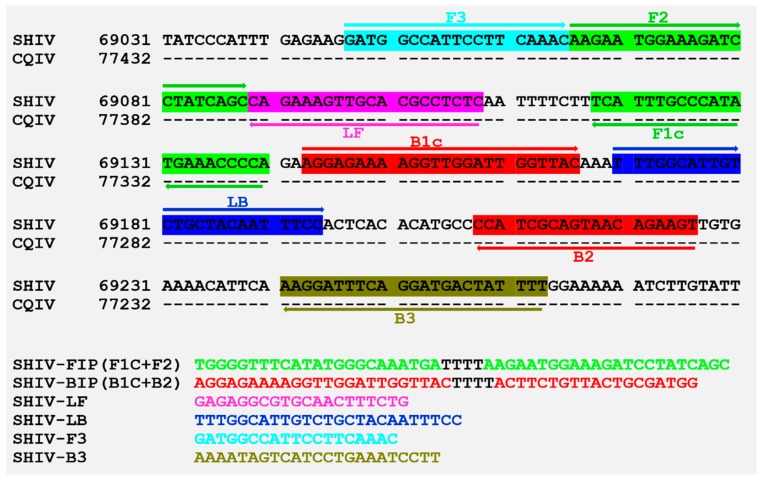
Information on primer design and primers used for Loop-mediated isothermal amplification (LAMP), based on the reference sequence of DIV1. Sequences of Shrimp hemocyte iridescent virus (SHIV 20141215 MF599468) and *Cherax quadricarinatus* iridovirus (CQIV CN01 MF197913) were obtained from GenBank.

Primers for LAMP were designed to target the gene of the second largest subunit DNA-directed RNA polymerase II located between 69047—69263 of SHIV 20141215 genome (GenBank access no. MF599468), of which the sequence was completely complementary to that of CQIV CN01 77416—77200 (Figure 5). In addition, results of LAMP primer specificity assay showed that the reactions were only positive for DIV1-infected shrimp, while the reactions were negative for WSSV, *Vp*_AHPND_, IHHNV, or EHP infected shrimps. The reactions indicated that the LAMP primers in this study were specific to DIV1 and should work for ISDL.

### 3.5. ISDL

**Figure 6:**
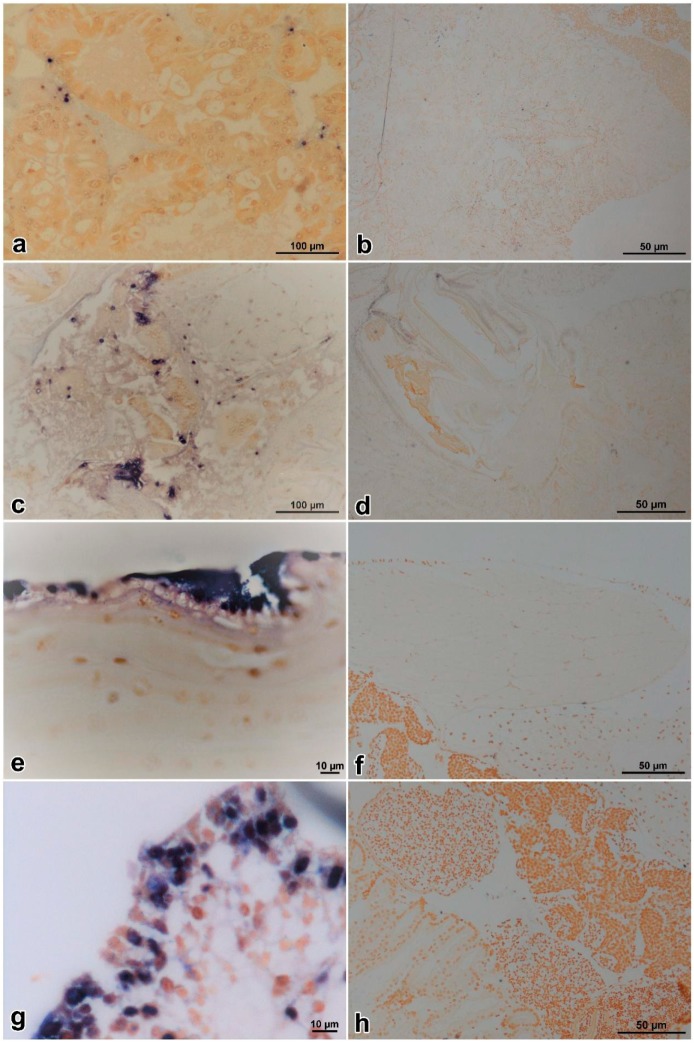
In situ DIG-labeling-loop-mediated DNA amplification (ISDL) micrographs of DIV1-infected and control *Exopalaemon carinicauda*. Hepatopancreas, stomach, cuticular epithelium, and hematopoietic tissue from a challenged prawn of the PO group, respectively (**a**, **c**, **e**, and **g**); hepatopancreas, stomach, cuticular epithelium, and hematopoietic tissue from a healthy prawn of the CPO group, respectively (**b**, **d**, **f**, and **h**). In pictures a, c, e, and g, blue-violet signals were observed in hepatopancreatic sinus, stomach epithelium, cuticular epithelium, and hematopoietic tissues of prawns, respectively. In pictures b, d, f, and h, no hybridization signal was seen in the same tissues of DIV1-negative *E. carinicauda*, except some non-specific signals on gastric sieve of stomach.

For ISDL protocol, a LAMP system was applied to in situ amplification to produce DIG-labeled products and subsequently detected with anti-DIG antibody on histological sections. ISDL results showed that blue-violet hybridization signals were detected in the hepatopancreatic sinus, stomach epithelium, cuticular epithelium, and hematopoietic tissues (Figure 6a,c,e,g) of prawns in the IM and PO groups, but no signal was detected in the control groups (Figure 6b,d,f,h). The results of ISDL provided further evidence that prawns in the IM and PO groups were infected by DIV1.

### 3.6. TEM

**Figure 7:**
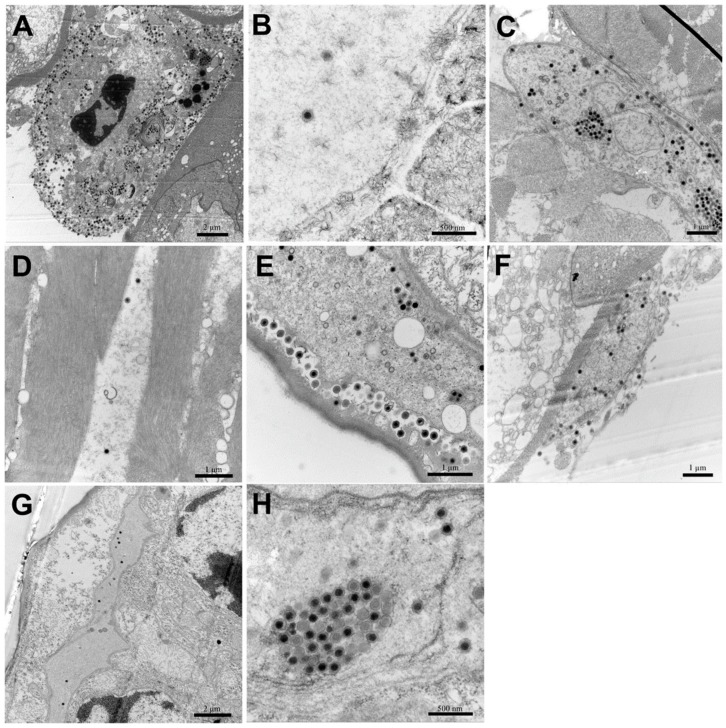
Transmission electron microscopy (TEM) of DIV1-infected *Exopalaemon carinicauda*. (**A**–**E**): hepatopancreatic sinus, pereiopods, uropods, muscle and gills, respectively, of a prawn in the IM group; (**F** and **G**): hepatopancreatic sinus and gills, respectively, of a prawn in the PO group; (**H**): cytoplasmic inclusions with a cluster of viral particles.

Icosahedral virions and cytoplasmic inclusions were observed in hepatopancreatic sinus, pereiopods, uropods, muscle, and gills of *E. carinicauda* of the IM group (Figure 7A–E,H). Assembled virions were mostly distributed outside of the assembling area near cellular membrane of the infected cells (Figure 7A,E). Similarly, virions were observed in the hepatopancreatic sinus and gills of prawns of the PO group (Figure 7F,G). Virions were hexagonal (i.e., icosahedral) and about 150 nm in diameter, which matched with characteristics of DIV1.

## 4. Discussion

The susceptibility of infected species and the host range for a newly found virus are important information for studying both host and virus. From the host aspect, knowing an infectious pathogen of a host species will provide important information for prevention of the relevant disease; from the virus aspect, knowledge of a susceptible host range can be used for identification of virus species, discovering a specific viral replication host or cell line, and understanding of viral ecology. Susceptible host range is also important for international trade to prevent the transboundary spreading of a specific disease. For such purpose, the OIE issued criteria in 2014 to determine susceptibility for specific diseases that requires evidence from either natural infection or experimental challenge mimicking natural infection [11]. As a newly found virus, the susceptible host range of DIV1 remains a largely unknown area to be investigated. For the natural infection pathway, positive detections of DIV1 by PCR or qPCR have been reported in different natural samples of crustacean, including *P. vannamei* [9], *P. chinensis* [9], *M. rosenbergii* [9,23], *C. quadricarinatus* [8], *Procambarus clarkii* [23,24], *P. japonicus* [23], *M. nipponense* [23,24], *M. superbum* [24], and Cladocera [24]. Severe disease with high mortality has been reported and infection with DIV1 has been demonstrated in *C. quadricarinatus*, *P. vannamei*, and *M. rosenbergii* [8,9,24]. All of these have been confirmed as being susceptible species for DIV1, fulfilling the natural infection criteria of OIE standards. The susceptibility of *P. chinensis*, *P. japonicus*, and *M. superbum* to DIV1 were not fully confirmed, as the evidence of PCR or qPCR positives [23,24] only supports the existence of DIV1. Confirmation of infection with DIV1 can usually be supported by evidence from histopathological evaluations, TEM, or in situ hybridization. However, they may remain suspicions of susceptibility, as there are confirmed susceptible species in the same genus. Cladocera has been detected as positive by qPCR, but the ISDL result was negative, so that it has been considered as a non-susceptible species [24]. For the experimental infection pathway, experimental challenges of *P. vannamei*, *Pr. clarkii*, Chinese mitten crab *Eriocheir sinensis*, and wild crab *Pachygrapsus crassipes* with CQIV CN01 have been reported [8,25]. Unfortunately, all these experimental infections only used only intramuscular injection, so the results were not enough to confirm of susceptibility for these species because OIE standards require that the challenge pathway to mimics natural infection. This present study is the first report to confirm a susceptible species to the newly found DIV1 by an experimental pathway mimicking the natural infection that fully follows OIE standards.

Due to an absence of a cell line for crustaceans, it is not possible to use normal plaque purification for crustacean viruses. We used a variety of methods to prove that *E. carinicauda* was successfully infected with DIV1, including molecular detection, histopathological and cytopathological analyses, and in situ DIG-labeling-loop-mediated DNA amplification. The original virus strain SHIV 20141215 has been demonstrated by testing for five major shrimp pathogens, including WSSV, YHV, TSV, IHHNV, and *Vp*_AHPND_ by PCR or RT-PCR methods [9]. In the previous study, the debris of *P. vannamei* and the purified viral inoculum were re-examined using real-time PCR for WSSV, TSV, YHV, IHHNV, *Vp*_AHPND_, CMNV, and DIV1 to exclude the possibility of contamination by other viruses and bacteria. In addition, no other viruses, with the exception of DIV1-like, as well as bacteria, were observed with TEM in samples used in this study.

Natively distributing in Yellow Sea and Bohai Sea and widely cultivated broadly in Jiangsu, Zhejiang, Shandong, and Liaoning Provinces of China, *E. carinicauda* is one of important economic crustaceans [12,13]. It has been reported that *E. carinicauda* is a natural host of some viral pathogens, such as WSSV [26] and CMNV [27]. *E. carinicauda* was also considered as a potential model animal for studies of shrimp viral pathogens [28].

In order to investigate the susceptibility of *E. carinicauda* to DIV1, following the OIE standards, the decapod was experimentally challenged with DIV1 per os and intramuscular injection. After 3 dpi, infected prawns of both the PO and IM groups displayed identical clinical symptoms, including debility, empty guts and stomach, pale hepatopancreas, slight whitish hematopoietic tissue, and death. Much higher doses of DIV1 were detected in hepatopancreatic samples of the moribund and deceased prawns in the IM group than those in the PO group. The significant difference in viral dose may be because the injection of DIV1 caused extensive and synchronous infection in cells of different target tissues. The clinical signs observed in *E. carinicauda* were similar to those of *P. vannamei* affected by SHIV 20141215 [9] and also similar to those of *C. quadricarinatus* affected by CQIV CN01 [8]. However, the sign of slightly whitish cloud at base of rostrum was not observed in decapods other than *M. rosenbergii*, which showed a typical white triangle at the hepatopancreatic locus [24]. The invisibility of this symptom in hematopoietic tissue of some DIV1 infected shrimp and crayfish may be due to interspersion and tininess of hematopoietic tissues of *Penaeus* species [18] or the opaque cuticles of crayfish. We may still need to search carefully for this symptom in future observation, as all gross signs in other tissues are not be typical for the infection with DIV1. According to the nonlinear regression of the challenge tests, in the duration of 16 dpi, mean cumulative mortalities of *E. carinicauda* were 70.8% and 42.5% caused by infection with DIV1 via intramuscular and per os, respectively. The pathogenic model (2) and (3) derived from the bioassay revealed that *E. carinicauda* showed some tolerance to the infection with DIV1 per os challenge mimicking natural infection. Compared with the intramuscular challenge, the per os challenge caused only (60.1 ± 6.3)% lower mortality within (145 ± 0.35)% longer time spent at (17.2 ± 14.0)% slower instant speed. The surviving DIV1 positive prawns in per os challenged group possessed viral loads at (3.57 ± 3.42) × 10^2^ copies/ng-DNA, which were about 100 folds lower than that detected in dead prawns. Previous studies showed that the mortalities of *P. vannamei*, *C. quadricarinatus*, and *Pr. clarkii* experimentally challenged with DIV1 reached 100% [8,9]. The partial tolerance of *E. carinicauda* to DIV1 may provide an object for future study on mechanisms and breeding of DIV1 resistance. As *E. carinicauda* is a broadly distributed native species, the partial tolerance of the ridgetail white prawn to DIV1 may also provide a possible reservoir of the virus after it transports to species in the area of the Yellow Sea, Bohai Sea, and East China Sea.

Positive results of TaqMan qPCR and ISDL provided further evidences of the infection of *E. carinicauda* by DIV1. According to the results of ISDL, positive signals were detected in the hepatopancreatic sinus, hematopoietic tissue, and cuticular epithelium of infected *E. carinicauda*. Previous studies showed that cuticular epithelium is a major target tissue of WSSV infection [26,29]. However, cuticular epithelium of *P. vannamei* is not a susceptible tissue to DIV1 infection. The results exhibit a difference in tissue tropism between *E. carinicauda* and *P. vannamei* to DIV1 infection. ISDL [19] is a highly sensitive and time-saving method, compared to normal in situ hybridization (ISH). In addition, the histopathology of diseased *E. carinicauda* was consistent or similar to that previously reported in *P. vannamei*, *C. quadricarinatus*, and *Pr. clarkii* [8,9]. Moreover, in this study, massive DIV1 virions with dense cores, characterized by icosahedral shape and measuring ~150 nm in diameter and consistent with the report of Qiu et al. [9], were observed in hemocytes of hepatopancreatic sinus and gills of *E. carinicauda* of the IM and PO groups. The virions primarily existed in cytoplasm of hemocytes and branchial cells. Additionally, in *E. carinicauda* of the IM group, DIV1 virions were also observed in pereiopods, muscles, and uropods. These results expanded the knowledge of tissue tropism of DIV1.

The combined results of clinical observation, molecular biological diagnosis, histological examination, and TEM supported the assertion that *E. carinicauda* is a newly confirmed susceptible species to infection with DIV1, following the criteria for listing species as susceptible to infection with a specific pathogen developed by the OIE [11]. Presently, there are no reports of natural infection of *E. carinicauda* with DIV1 in a farm or in the sea. As *E. carinicauda* has local economic importance, biosecurity strategies [30,31] should be taken into account, considering the risk that DIV1 can cause disease in *E. carinicauda* and the risk that the species may become a possible reservoir of DIV1. It also should be ignored that *E. carinicauda* showed some degree of tolerance to infection with DIV1, especially per os challenge mimicking a natural pathway.

## Supplementary Materials

The following is available online at https://www.mdpi.com/1999-4915/11/4/387/s1, Figure S1: Positive data of DIV1 qPCR detection within 40 cycles in IM and PO groups.
