# Supplementary figures and images for: Susceptibility of Exopalaemon carinicauda to the Infection with Shrimp Hemocyte Iridescent Virus (SHIV 20141215), a Strain of Decapod Iridescent Virus 1 (DIV1)
Source: Viruses. 2019 Apr 25;11(4):387. doi: 10.3390/v11040387 (PMC6520858; doi:10.3390/v11040387)

## Supplementary

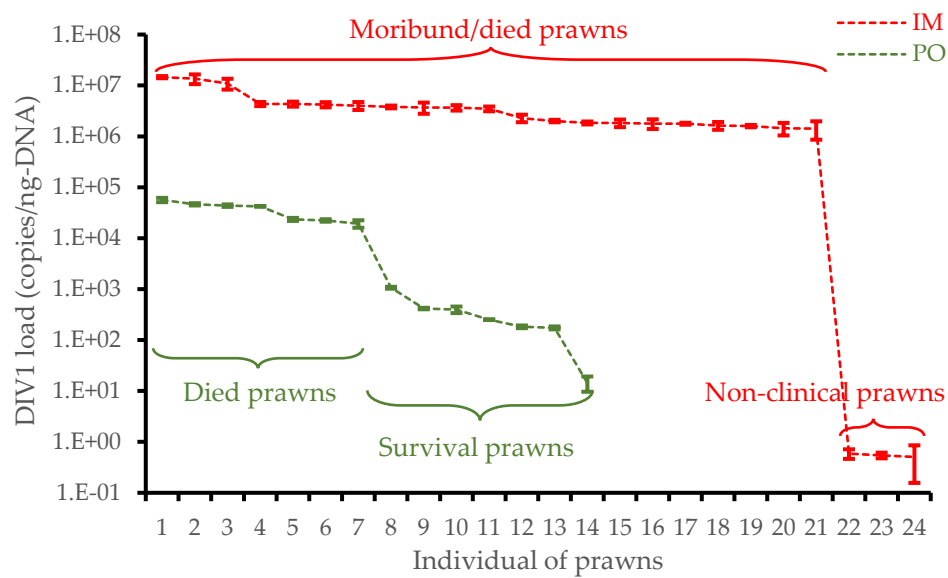

**Figure S1.** Positive data of DIV1 qPCR detection within 40 cycles in IM and PO groups.

Supplement: Supplementary file 1 [file viruses-11-00387-s001.pdf]
